# Huge decrease of frost frequency in the Mont-Blanc Massif under climate change

**DOI:** 10.1038/s41598-019-41398-5

**Published:** 2019-03-20

**Authors:** Benjamin Pohl, Daniel Joly, Julien Pergaud, Jean-François Buoncristiani, Paul Soare, Alexandre Berger

**Affiliations:** 1 Biogéosciences, UMR6282 CNRS/université Bourgogne Franche-Comté, Dijon, France; 2 ThéMA, UMR6049 CNRS/université Bourgogne Franche-Comté, Besançon, France

## Abstract

Mountains are a sensitive indicator of climate change and these areas are an early glimpse of what could happen in lowland environments. Peaking at 4808 m asl, the Mont-Blanc summit, at the boundary between France and Italy, is the highest of the Alps, in Western Europe. Its Massif is world-famous for outdoor and extreme sport activities, especially since the 1924 Olympic games held in Chamonix. Here, we use a novel statistical downscaling approach to regionalize current and future climate change over the Mont-Blanc Massif at an unequalled spatial resolution of 200 m. The algorithm is applied to daily minimum and maximum temperature derived from global climate models used in the fifth assessment report of the International Panel on Climate Change (IPCC). This new high-resolution database allows for a precise quantification of frost occurrence and its evolution until 2100. In the winter season and by the end of the 21^st^ century, under a pessimistic scenario (RCP8.5), frost frequency in the morning could decrease by 30–35 percentage points in the valley of Chamonix, and in the afternoon, similar changes could occur for elevations comprised between 2000 and 3000 m. In summertime, changes are even larger, reaching a huge drop of 45–50 points in the afternoon between 3500 and 4500 m. These changes are much reduced under an optimistic scenario. They could have huge impacts on the environment (glacier shrinking, permafrost degradation, floods, changes in the distribution of species and ecosystems) and societies (summer tourism for climbing and hiking, and winter tourism for skiing).

## Introduction

**Figure 1:**
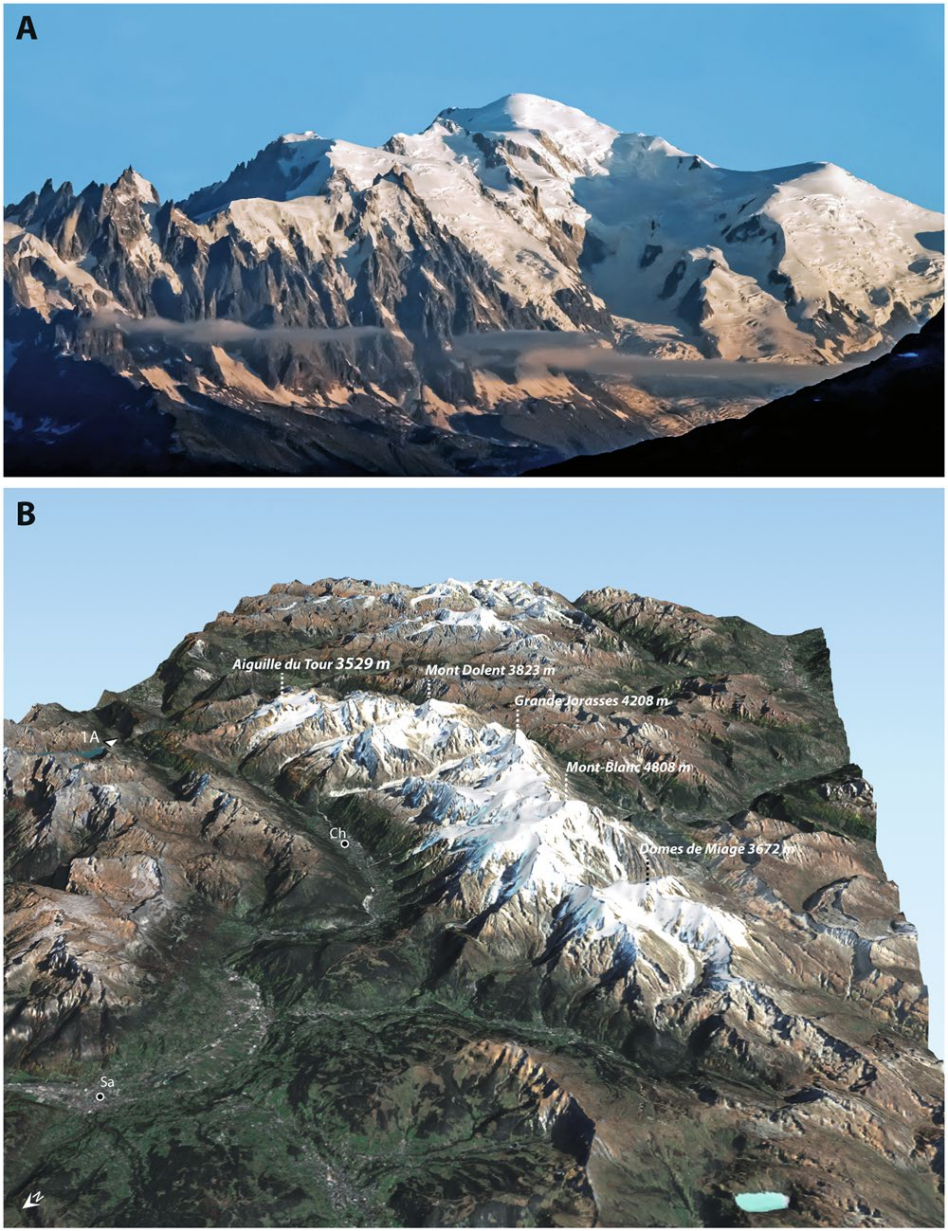
Presentation of the Mont-Blanc Massif. (**a**) The Mont-Blanc summit seen from Emosson Dam (French-Swiss border). (**b**) 3D view of the Mont-Blanc Massif.

The vulnerability of temperate mountain ranges to global warming is partly related to their elevation^1,2^, steep slopes and orientation towards the sun^1^. The Mont-Blanc massif is one of the highest in the Alps (Fig. 1): 38 km^2^ of its surface shows elevations of at least 3500 m, 8 km^2^ peak above 4000 m but only 1 km^2^ above 4500 m. This raises the question of the availability of so-called climatic shelters, that is, areas where the ecosystems can migrate to remain in environmental conditions favourable to their development^3^. In the plains of the mid-latitudes, a rise of 0.6 °C typically corresponds to a poleward shift of one latitude degree (about 111 km). In the temperate mountain ranges, this corresponds to a vertical shift of 100 m only^4^. A warming of several Celsius degrees, as estimated by the fifth assessment report of the IPCC, could thus lead to a general migration of several hundreds of meters vertically, and cause a dramatic transformation of high-mountain environments^2^. In high elevations, temperature remains negative almost constantly, favouring snow accumulation and permafrost in non glaciated areas^5,6^. This is likely to change in the future, hence the need for very high-resolution climate projections.

Regionalizing climate change^7,8^ in mountain regions is a challenging task, because reliefs are drastically smoothed in the coarse grids of current climate models^9,10^. At spatial resolutions of a few tens of kilometres, the Alps barely reach 2000 or 2500 m above sea level, which is not suited to estimate the interaction between the topography and climate variability and change in these regions^11,12^. Statistical downscaling algorithms^13,14^ (see Supplementary Information) are appropriate tools to estimate the spatial variability of variables like air temperature, using spatial predictands derived from high-resolution digital elevation models, and large-scale temperature changes (predictors) taken from climate models. Here, we use a novel algorithm, allowing for more precise and refined temperature estimations^4^, to regionalize climate change as simulated by a sample of 13 climate models (Supplementary Table 1) at a spatial resolution of 200 m. The two most contrasted greenhouse gas emission scenarios are selected, namely RCP2.6 (optimistic) and RCP8.5 (pessimistic), to consider the whole spread of possible climate evolutions, together with historical simulations conducted with observed concentrations of greenhouse gases since the 1850s. This downscaling procedure is necessary to consider the combined effects of elevation, slopes, orientation and valleys, on daily minimum (Tn) and maximum (Tx) air temperature. In the resulting elevation model (Fig. 1b), the Mont-Blanc summit peaks at 4763 m instead of 4808 m in the real world, illustrating the high degree of realism reached by such hectometric resolution.

## Frost Occurrence: a Relevant Metric for Impacts on Mountain Environment and Societies

**Figure 2:**
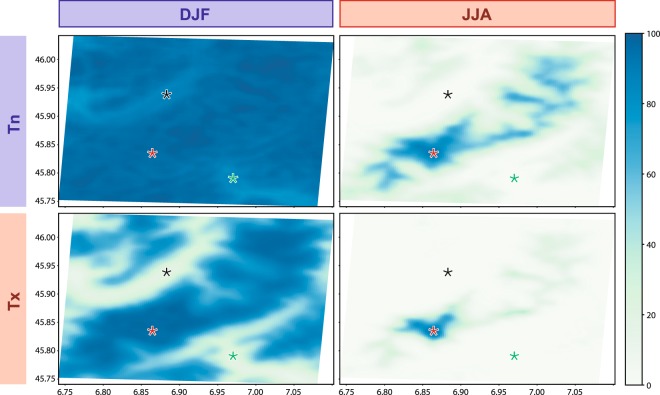
Average frost occurrence (%, see colorbar) for Tn and Tx and for summer and winter seasons under current climate conditions (period 1979–2014) according to spatially interpolated observations. The black star corresponds to the Chamonix Mont-Blanc station in France (1035 m), the red star to the Mont-Blanc summit (4810 m) and the green star to the Courmayeur station in Italy (1224 m).

We focus here on frost frequency evolutions, since this metric is of major importance for the rain-snow line, glacier melting or ice accumulation, the hydrology of the region and rock wall erosion though cryoclasty. Variability and long-term changes in frost frequency are thus accompanied by dramatic effects on the environment^3^, landscape^15,16^, and human activities^17,18^. Under current climate conditions (Fig. 2), in the morning (Tn) during the winter season, the 0 °C threshold is almost never exceeded, except in the bottom of the lower valleys like Chamonix or Courmayeur (<1200–1500 m). In the afternoon (Tx), the air mass warms and the occurrence of frost strongly decreases under 1500 and 2000 m asl. In summer frost is circumscribed to higher elevations, and frost predominates above 2500–3000 m asl for Tn and 3500–4000 m asl for Tx. These estimates (obtained as multiple regression based on observed temperature measurements^4^: the “interpolation” step^4^), are associated with very weak errors and uncertainties, and can thus be considered as reliable.

**Figure 3:**
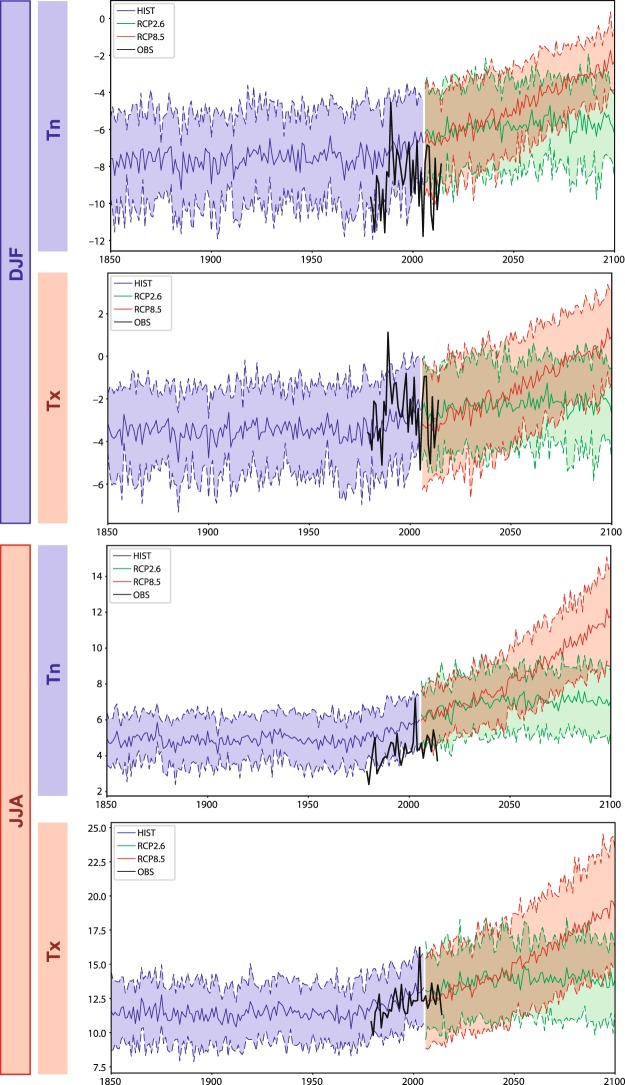
Spatially-averaged temperature evolutions over the Mont-Blanc Massif for Tn and Tx and for summer and winter seasons. The spatial domain used for computation is that shown in Fig. 2. Black curves: spatially interpolated observations, period 1979–2014. Blue colours: historical (HIST) simulations, period 1850–2005. The solid curve shows the ensemble mean, the colour shading extends to ± 1 standard deviation to show model uncertainties. Green colours: the same for RCP2.6 simulations, period 2006–2100. Red colours: the same for RCP8.5 simulations, period 2006–2100.

The next step consists in applying the same spatial models to downscale climate change simulations (the “downscaling” *per se*^4^). In such temperature estimates, the spatial variability is thus inherited from the statistical laws deduced, under current climate conditions, from the aforementioned interpolations, while the large-scale temporal variability of air temperature is derived from the global models involved in the Coupled Model Inter-comparison Project phase 5^19^ (CMIP5). Using the same 200-m horizontal projected grid over the Mont-Blanc Massif and its surroundings, summer (JJA) and winter (DJF) mean temperature evolutions from 1850 to 2100 are compared to interpolated observations (Fig. 3). Our data clearly show the heat wave for summer of 2003^20^, while the trimester of DJF 1989–1990 holds the record for winter temperature over the period. Over their common period, the difference between observations and historical simulations allows for an estimate of the typical errors of climate models over this region (Supplementary Figure 2). Models over-estimate Tn both in summer and winter, and produce much more realistic results for Tx estimates. The amplitude of the future warming is strongly dependent on the emission scenario. Optimistic RCP2.6 produces stabilized temperatures at about +1.5 °C compared to pre-industrial levels: it is the only scenario that meets the terms of the Paris Agreement on climate change locally, that is, a “temperature rise this century well below 2 Celsius degrees above pre-industrial levels”. Pessimistic RCP8.5 leads to a warming of roughly +2 °C in wintertime (+2.8 °C in summertime) by the mid-century, these values reaching respectively +3.8 °C and +5.6 °C for the late century. Although all downscaled models produce qualitatively similar results, uncertainties remain large, as denoted by the inter-model spread in Fig. 3. For the late 21^st^ century for instance, and from one model to another, summer temperature rise is comprised between +5 and nearly +9 °C for Tx (+4 °C to +7.5 °C for Tn). Depending on the models, the record heat wave of 2003 could become representative of a typical summer season between 2060 and 2075. The abnormally warm winter of 1989–1990 could represent the typical winters of the years 2070–2080.

## Evolution of frost occurrence by the mid- and late-century

**Figure 4:**
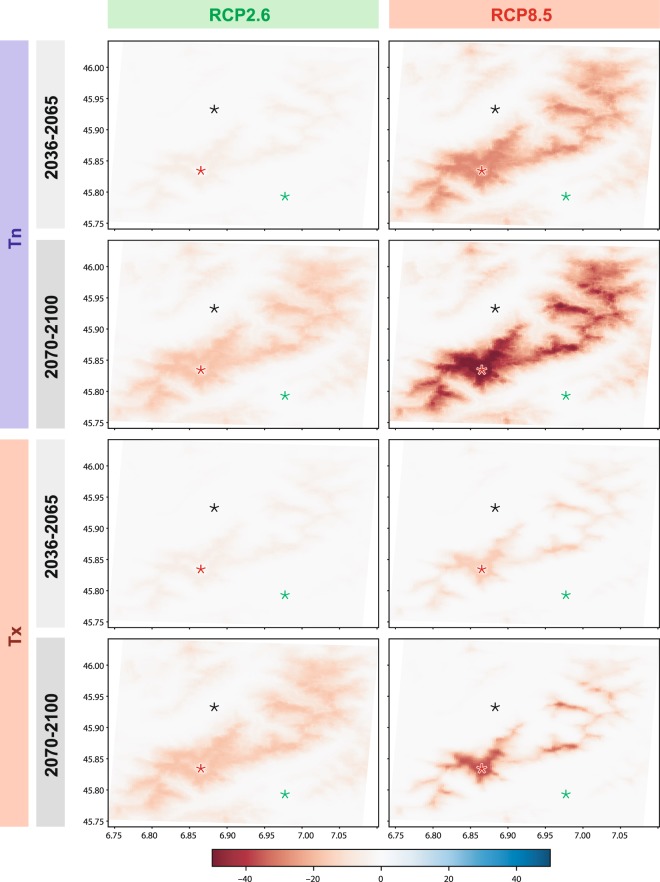
Evolution of frost occurrence (percentage points) for the summer season. Maps represent the difference between RCP and HIST simulations (averaged over period 1970–2000) for mid-century (period 2036–2065) and late century (2070–2100). Maps are multi-model averages for Tn and Tx and for RCP2.6 and RCP8.5.

Results are next refined at the daily timescale, through an analysis of daily frost occurrence, to represent the evolution in the summertime frost (Fig. 4). This is done under contrasted emission scenarios and for the mid- and late century, compared to current climate conditions (as described by historical simulations). Errors inherited from the CMIP5 are shown in Supplementary Figure 2, corresponding uncertainties in Supplementary Figure 3, and similar analyses applied to the winter season in Supplementary Figure 4. Projected changes are of larger magnitude for Tn, because most of the domains already experiences positive temperature in summer under current climate conditions (Fig. 2). They are much sensitive to the emission scenario, especially for the late century. Morning frost occurrence (Tn) decreases by about 25–30 pp over most of the Mont-Blanc massif in the mid-century under RCP8.5, and slightly less than that for the late century under RCP2.6. Frost occurrence experiences a huge drop of 45–50 pp for the late century for RCP8.5. The geography of these changes is strongly driven by the topography (Fig. 1). Crucially, even the Mont-Blanc summit shows obvious evolutions: up to −20 pp by the end of the century for frost occurrence in the morning (Tn), and even –35 pp in the afternoon (Tx), according to the RCP8.5 scenario. Such decrease may lead to major changes of its local climate. Under current climate conditions, it has only experienced brief thaw events^21^, like during the heat wave of summer 2003. This could favour negative mass balance for most glaciers^22^, due to drastically enhanced melting and decreased accumulation in high elevation environments.

**Figure 5:**
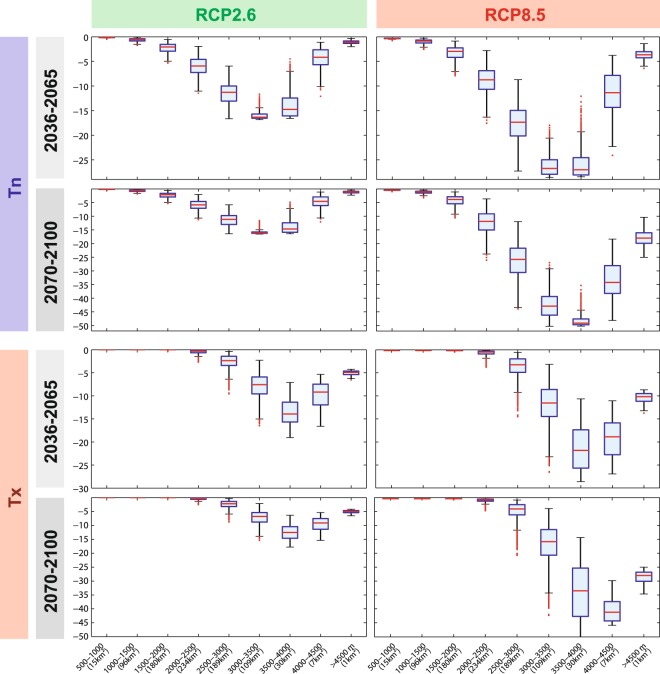
Boxplot representation of the evolution of frost occurrence (percentage points) for the summer period, according to elevation. Analyses are the same as the maps of Fig. 4. The boxes have lines at the lower, median and upper quartile values. The whiskers are lines extending from each end of the box to 1.5 interquartile range. Plus signs correspond to statistical outliers.

The present altitudinal range of frost occurrence will show major changes during the 21st century (Fig. 5). Main changes occur between 2000 and 4000 m asl for RCP2.6 in the morning (Tn), and between 3000 and 4500 m in the afternoon (Tx), according to RCP2.6 downscaled simulations. They are much higher in elevation and magnitude for RCP8.5 (up to −45–50 pp between 3500 and 4000 m asl for Tn and −30 to −50 pp at the same elevation for Tx by the late century).

These evolutions in the climate of the Massif are likely to induce major changes in the dynamics of the snowpack^23^, cryosphere^24^, hydrosphere^25^ and biosphere^15^. They could lead to major, but complex changes in hydrological regimes, especially in the upper alpine valleys, through changes in the relative contribution of their glacial and nival components^26^. Increased frequency in frost-thawing alternations could also enhance gelifraction, which, together with degradations in the permafrost^18,27^, could increase rockfall occurrence^28–30^ in the future. These changes might also force human activities to adapt to such shifts of the isotherms and rain-snow line. Major impact are expected on winter sports, leading ski resorts^31,32^ to higher elevations in parallel with the migration of biomes^33^ and environments. Given the narrowness and vulnerability of high mountain environments, this could potentially lead land-use conflicts. Increased anthropogenic pressure on these vulnerable areas has the potential to further perturb their fragile ecosystems, highlighting the need for sustainable development and planning strategies.

## Methods

A frost event is defined as a day for which Tn < 0 °C for morning frost, or Tx < 0 °C for afternoon (permanent) frost. Frost frequency (in %) is obtained as the ratio between the number of frost events and the total length of the period. Frost evolutions under climate change consist in differences between future and current frost frequencies and are thus expressed as percentage points (pp). The statistical downscaling^4^, as used here, combines temporal variability derived from CMIP5 simulations, and spatial variability based on statistical relationships obtained on observed time series (an original database of 60 weather stations available daily over a 35-year period). Temperature biases are inherited from both the CMIP5 model and the statistical downscaling: see Supplementary Information and methodological paper^4^ for details. Uncertainties arise from disagreements between CMIP5 models, hence the use of 13 different simulations for robust convergence assessment.

## Supplementary information

Supplementary Information
