# Supplementary material for: Huge decrease of frost frequency in the Mont-Blanc Massif under climate change
Source: Sci Rep. 2019 Mar 20;9:4919. doi: 10.1038/s41598-019-41398-5 (PMC6427016; doi:10.1038/s41598-019-41398-5)
Supplement: Supplementary file 1 — Supplementary Information [file 41598_2019_41398_MOESM1_ESM.docx]

Huge decrease of frost frequency in the Mont-Blanc Massif

under climate change

Benjamin Pohl, Daniel Joly, Julien Pergaud, Jean-François Buoncristiani, Paul Soare, Alexandre Berger

**Supplementary Information**

**Supplementary Methods**

**1. CMIP5 Earth System Models**

In this paper we selected 13 model versions out of the 39 reported in the fifth Assessment Report of IPCC^1^. This choice is motivated by various factors: (i) we tried to respect an “institutional democracy”^2^ among the modeling groups that participated to the CMIP5 exercise; (ii) we selected all the models for which the requested simulations (historical, RCP2.6 and RCP8.5) and variables (daily Tn, Tx and precipitation amounts) were archived at the daily timescale and accessible online. **Supplementary Table 1** summarizes the model versions used here, together with the institutions in charge of the model developments. Are also reported: the elevation of the closest grid point to the Mont-Blanc Massif, and the periods over which the model outputs were downscaled for the three radiative forcings used in this work (HIST for historical simulations, RCP2.6 and RCP8.5 for climate projections by the end of the 21^st^ century). More detailed information concerning these models can be found in the fifth Assessment Report of IPCC^1^ (Working Group I, Chapter 9 ). These data are next used as predictors assessing large-scale temperature variability and change and are combined with predictands derived from the topography to estimate the spatial variability of temperature over and near the Mont-Blanc Massif.

| **Model Acronym** | **Institution (Country)** | **Elevation**  **(m)** | **HIST** | **RCP26** | **RCP85** |
| --- | --- | --- | --- | --- | --- |
| **BCC** | Beijing Climate Center (China) | 780 | 1850-2012 | 2006-2105 | 2006-2300 |
| **BNU** | Beijing Normal University (China) | 780 | 1950-2005 | 2006-2100 | 2006-2100 |
| **CanESM2** | Canadian Centre for Climate Modelling and Analysis (Canada) | 780 | 1850-2005 | 2006-2100 | 2006-2100 |
| **CNRM** | Centre National de Recherches Météorologiques (France) | 1764 | 1850-2005 | 2006-2100 | 2006-2100 |
| **CSIRO** | Commonwealth Scientific and Industrial Research Organisation (Australia) | 1473 | 1970-2005 | 2006-2100 | 2006-2300 |
| **FGOALS** | Institute of Atmospheric Physics, Chinese Academy of Sciences and Tsinghua University (China) | 488 | 1950-2005 | 2006-2100 | 2006-2101 |
| **GFDLM2M** | Geophysical Fluid Dynamics Laboratory (USA) | 1226 | 1861-2005 | 2006-2100 | 2006-2100 |
| **IPSL-CM5A-LR** | Institut Pierre-Simon Laplace (France) | 1161 | 1950-2005 | 2006-2300 | 2006-2300 |
| **IPSL-CM5B-MR** |  | 1336 | 1850-2005 | 2006-2100 | 2006-2100 |
| **MIROC5** | Atmosphere and Ocean Research Institute / National Institute for Environmental Studies, and Japan Agency for Marine-Earth Science and Technology (Japan) | 502 | 1850-2012 | 2006-2100 | 2006-2100 |
| **MPI-MR** | Max Planck Institute for Meteorology (Germany) | 887 | 1850-2005 | 2006-2100 | 2006-2100 |
| **MRI** | Meteorological Research Institute (Japan) | 1926 | 1850-2005 | 2006-2100 | 2006-2100 |
| **NOR** | Norwegian Climate Centre (Norway) | 1293 | 1850-2005 | 2006-2100 | 2006-2100 |

**Supplementary Table 1 |** Summary of the model versions (such as they appear in the fifth Assessment Report of IPCC, first column), associated institutions and countries (second column), the elevation of the closest grid-points at which the data were retrieved and selected as predictor for the statistical downscaling procedure (column 3), and the years over which daily Tn and Tx values were extracted for this work for historical simulations (HIST, column 4) and contrasted RCP climate projections (columns 5 and 6).

**2. Geomatic Downscaling: General principles and application to CMIP5 simulations**

The algorithm of the so-called geomatic downscaling is extensively presented in a dedicated methodological paper^3^. It basically consists in a statistical downscaling procedure, that uses various predictands derived from the topography, and a predictor taken from coarse-resolution datasets representative of the regional-scale climate (e.g., CMIP5 numerical simulations). It includes a mathematical formalism considering both the frequency at each predictand is selected, and its specific contribution to explain the spatial variability of a given geophysical field. These refinements allow for an improvement of the estimations (that is, a decrease in associated errors) by typically 10% compared to other algorithms solely based on regressions with elevation and other derived variables.

The algorithm proceeds as follows.

First, it separated the temporal and spatial variability of the geophysical field to be estimated (air temperature in our case). Temporal variations are taken from the grid-point of each CMIP5 model outputs which is closest to the Mont-Blanc Massif. Because each model has different grid geometries, including very different resolution and grid-point locations, the elevation of the selected grid-points are highly variables from one model to another (**Supp. Table 1**), making direct comparisons of raw model outputs uneasy. The statistical downscaling procedure^3^ offers the first advantage to project these data onto the same 200-m resolution grid, hereby making the model outputs inter-comparable whatever their native grid. To that end, raw temperature values extracted from the CMIP database are first brought onto a standard reference elevation of 0m, using an average environmental lapse rate of –0.6°C / 100m. Empirically, the environmental lapse rate as measured by weather stations is slightly lower from the theoretical –0.6°C / 100m used here^3,4^. This is due to thermal inversions in the valleys, which are quite common in the Alps (and may cause air quality issues, especially in the Chamonix valley). Yet, CMIP models often fail at reproducing such inversions, partly in line with their too coarse horizontal and vertical resolutions. Thus, in the models, this is the theoretical lapse rate that prevails, and that should be used to correct altitudinal differences between the model grids.

The same transformation is next applied the other way around, by bringing each temperature value up to the average elevation in the domain of interest, such as it appears in the 200-m resolution digital elevation model (DEM). It is noteworthy that this DEM is derived from native databases available at a 25-m resolution, which were then re-projected onto a regular 200m x 200m grid. Such resolution appears as a reasonable compromise between the realism of resulting topography, and the amount of data produced.

At the finer spatial scale of the Mont-Blanc Massif, temperature shows huge spatial variability, which is mostly related to surface conditions (including topography: elevation, exposure, slope, …)^5^. In the geomatic downscaling procedure, the method used to assess the influence of topography on the geophysical variable to estimate uses multiple linear regressions, combined with the frequency at which each environmental predictand is significant^3^. Such regression coefficients and frequencies are determined using observations. To do so, an original database of daily minimum and maximum temperature records has been employed, based on weather stations located in France, Switzerland and Italy at less than 100km from the Mont-Blanc Massif. Nearly 60 stations^3^, the elevation of which ranged between 235 and 3842m above sea level, have been used over a 35-year-long period (1979-2014) to constraint the statistical relationships that link temperature to its environmental predictands. Such coefficients and frequencies are computed separately for each variable (minimum and maximum temperature) and for each month of the year, allowing for a differentiated influence of each predictand on each variable over the annual cycle.

The final step consists in merging, for each day, each scenario and each model, the signals derived, on the one hand, from the CMIP5 predictor (temporal variability), and on the other hand, from spatial predictands based on the topography (spatial variability). This is achieved by computing the sum of (i) the temperature obtained by the GCM, and brought at the average elevation of the domain considered in the study; and (ii) for each topographic predictand, the product of the regression coefficient by the static value of each 200-m pixel in the domain. More details, as well as error and uncertainty assessment, are given in the dedicated methodological paper^3^.

For the present study, we considered 13 CMIP5 models under 3 radiative forcings (HIST, RCP2.6 and RCP8.5) and two variables (Tn and Tx), giving a total of 4,555,307 virtual days. Considering the size of the domain encompassing the Mont-Blanc Massif (containing 21,546 pixels at a 200-m resolution), this gives a grand total of nearly 98.15 billions of temperature estimates.

**3. Geomatic Downscaling: Selection of Predictors**

While the choice of the predictands is extensively discussed in the methodological paper^3^, the application of the downscaling algorithm to CMIP5 climate projections raises the question of the robustness of the predictor. Retaining solely the nearest grid-point of each climate model may be discussed, since this choice assumes that a single time series may be considered as representative of the time evolution and variability of the whole large-scale (that is, regional-scale) climate. Previous work reported that non-negligible uncertainties may come from such assumption^6^. Here, this issue is addressed by applying the same procedure not only on the grid-point closest to the Massif, but also on its surrounding eight neighbours. We use thus the 9 time series corresponding to the 9 nearest grid-points, considered sequentially as “potential predictors” for our downscaling procedure. The aim is to assess to which extent a single grid-point gives a coherent and representative view of the regional-scale climate variability and changes, when compared to its neighbouring grid-points. This allows for a precise quantification of the uncertainties associated with the choice of the predictor, which can next be compared to those inherited from the model-to-model differences. Results are shown in **Supplementary Figure 1**.

The seasonal mean temperature as derived from historical simulations shows non-negligible model-dependency, illustrating that the CMIP5 models already show some spread in their steady state over the past decades. From one grid-point to another, the spatial variability around the Alps is also model-dependent, some models producing more uniform, less variable seasonal mean temperature fields around the domain. The behavior of each model is generally consistent for both seasons and both variables: for instance, BCC and FGOALS systematically produce highly variable temperature fields around the Alps, thereby contrasting with MIROC and MPI which are always more uniform spatially. Importantly, the central grid-point (used for this paper) generally takes a median position, while grid-points located south of it tend to be warmer. Part of this spread is thus related to the zonal gradient of temperature in the mid-latitudes. Analyses of variance, used to test the ratio between intra-model and inter-model variance, assess that the models discriminate overall variance at a significance bound exceeding 99.9%. This means that the main uncertainties are more related to the differentiation between models, than between grid-points. As far as the seasonal mean temperature is concerned, considering a large ensemble of CMIP5 models with only the nearest grid-point used as predictor is thus sufficient to have a satisfactory view of the main source of uncertainties in the ensemble.

The same approach is used for the time evolution of seasonal mean temperature between the historical period and the mid- and late century, according to both the RCP2.6 and RCP8.5 radiative forcings. Once again the results are strongly model-dependent: each CMIP5 model has a specific response (referred to as climate sensitivity in the literature) to prescribed evolutions of greenhouse gases, a result well documented at the global and regional scales^7–9^ and characterized here at the local scale. As for the seasonal mean temperature, simulated warming over the current century and associated uncertainties are significantly assessed when considering solely the nearest grid-point as predictor: this result, obtained once again through the analyses of variance, assess that intra-model variability is of lesser importance than inter-model variability. This is especially true for the late century and RCP8.5, and slightly less robust, statistically speaking, for mid-century periods under RCP2.6. For all periods and RCP, it can nonetheless be concluded that extracting one single grid-point per model can be sufficient, in our case, to represent the regional climate mean state and long-term evolutions. Yet, considering a large number of model simulations is strongly recommended for robust uncertainty assessment.

**🡸 Supplementary Figure 1 |** Sensitivity to the predictor: mean climate and evolution throughout the 21^st^ century as derived from the 9 grid-points of each CMIP5 model nearest to the Mont-Blanc Massif. Upper panels: seasonal mean Tn and Tx in winter (DJF) and summer (JJA) seasons in historical simulations (HIST), spatially averaged over the whole domain after considering the mean altitudinal gradient (see text for details). The red circle corresponds to the nearest grid-point used as predictor in the remainder of this study. Blue circles correspond to the surrounding 8 grid-points (dark blue circles are located further north and light blue further south: see Legend in the figure). The estimated significance of an analysis of variance testing whether climate models discriminate the seasonal mean temperature is labelled on each panel of the figure. Lower panels (from top to down): as upper panels but for temperature changes (future period minus historical simulations) according to RCP2.6 (mid-century and late century) and RCP8.5 (mid-century and late century). Temperature differences are calculated separately for the 9 considered grid-points of each climate model.

As a complementary initiative to downscale climate projections over Europe, the Euro-CORDEX database^10^ could also have been considered in this study. The targeted resolution is however sensibly lower (0.11° at best), because Euro-CORDEX covers the whole European subcontinent. Hence, statistical / geomatic downscaling of Euro-CORDEX outputs would still be needed, hereby reducing the interest of this database since it could merely be used to provide large-scale predictors. Since there are fewer models than in CMIP5, used in a different methodological framework making them hardly comparable with global Earth System Models, we chose not to include Euro-CORDEX predictors in this study. The usefulness and the relevance of this database to regionalize climate projections and changes at larger scales, e.g. over the whole Alps, should nonetheless be acknowledged.

**Supplementary Results**

**1. Model error and uncertainty assessment**

The downscaling procedure is necessary for computing and comparing Tn and Tx time evolutions taken from various climate models at various resolutions, such as presented in **Fig. 3** of the main paper. This is because each model has a different resolution and grid, which implies that their raw outputs cannot be directly compared over a small territory such as the Mont-Blanc Massif (topography being more smoothed in coarser-resolution models). By projecting all grids onto the same 200-m resolution digital elevation model, the geomatic downscaling makes all models comparable, whatever their original grids. This step is equally necessary for analysing the frequency at which a fixed threshold (e.g. the 0°C isotherm) is reached at each location. Such analyses are applied to each climate model (**Supp. Table 1**), allowing for a detailed assessment of their mean response to radiative forcing scenarios, but also their disagreements (also shown in **Supp. Fig. 1**), quantified here as the inter-model standard deviation (**Fig. 3**).

Comparison between the downscaled historical simulations and interpolated observations during the same years (and thus under similar radiative forcings) allow for an assessment of the model biases, regardless their native resolution and grid. Tn and Tx biases are discussed in the main paper, and can be visually identified in **Fig. 3**. While biases induced by the statistical downscaling procedure are extensively discussed in the methodological paper presenting this algorithm^3^, errors originating from the local extractions of the CMIP model outputs are of about +2°C for Tn estimates in winter, and +1.5°C in summer. Their absolute value is weaker than 0.4°C for Tx estimates (**Fig. 3**). We address here the important issue of the effects of these biases for frost occurrence assessment.

Due to such warm bias of climate models for Tn (**Fig. 3**), current frost occurrence is under-estimated in HIST simulations, which results in under-estimating the long-term evolutions in frost frequency in the corresponding elevations. In winter, morning frost occurrence is under-estimated in the bottom of the Chamonix and Courmayeur valleys (**Supplementary Figure 2**). Similar results are found for elevations comprised between 2000 and 3000m for Tx, that is, close to the 0°C isotherm and the maximal gradient of frost frequency under current climate conditions (**Fig. 2** of the main paper). Other errors of secondary importance can also involve daily temperature variability or the downscaling procedure. Errors in Tx being much weaker on average (**Fig. 3**), frost occurrence is also more realistically in the afternoon (**Supplementary Figure 2**). Remaining errors are attributed (i) to spurious day-to-day variability in simulated temperature, leading to wrong probability density functions of daily temperature, these biased fluctuations, very model-dependent, causing errors in the frequency of frost (not shown); (ii) to the downscaling algorithm itself, especially in highest elevations (>3800m) where there are no long-term observations to constraint the statistical model. Combining with the high elevation of the 0°C isotherm during the summer season, this could explain the non-negligible errors found around the Mont-Blanc summit for afternoon frost regionalisation in JJA (**Supplementary Figure 2**).

Considering the usual environmental lapse rate of –0.6°C per 100m of elevation that prevails in the free atmosphere, Tn errors correspond to a vertical shift of about by 200 to 350m. These values should be taken as the typical error produced by the combination of the CMIP5 model biases and statistical downscaling procedure. Hence, the discretization of frost evolution throughout the century in **Figure 5** and **Supplementary Figure 5** uses 500-m elevation bins, a value fine enough to depict the vertical profiles in detail, but with a magnitude close to the typical errors noted above for morning frost estimations.

Beside the crucial question of the model errors, **Supplementary Figure 3** shows the model uncertainties, that is, the typical disagreement that one can expect from one model to another inside the selected CMIP ensemble presented in **Supplementary Table 1**. While the main paper presents the current frost frequency according to interpolated observation, and then its long-term evolution in the CMIP multi-model ensemble mean, **Supplementary Figure 3** presents raw frost frequency under current and future conditions, according to the multi-model ensemble mean but also with associated model spread. The latter is here quantified through the inter-model standard deviation. Results show (i) general uncertainties comprised between ~ 5 and 30pp, and which remain approximately constant in magnitude over the 21^st^ century; (ii) a clear tendency for larger uncertainties to concentrate close to the 0°C isotherm, that is, the altitudinal location of the sharpest gradient of frost frequency (**Fig. 2**, **Supp. Fig. 3**). These uncertainties relate to model biases in the average temperature simulated for the summer season (as shown in **Fig. 3**) but also to biased variability at the daily timescale around this mean steady state (see discussion above). These uncertainties, of moderate amplitude, lead to the same qualitative conclusions locally as those obtained at the regional and even global scale, that is, that the model uncertainties remain larger than the long-term changes for the first half of the century, while the hierarchy changes thereafter.


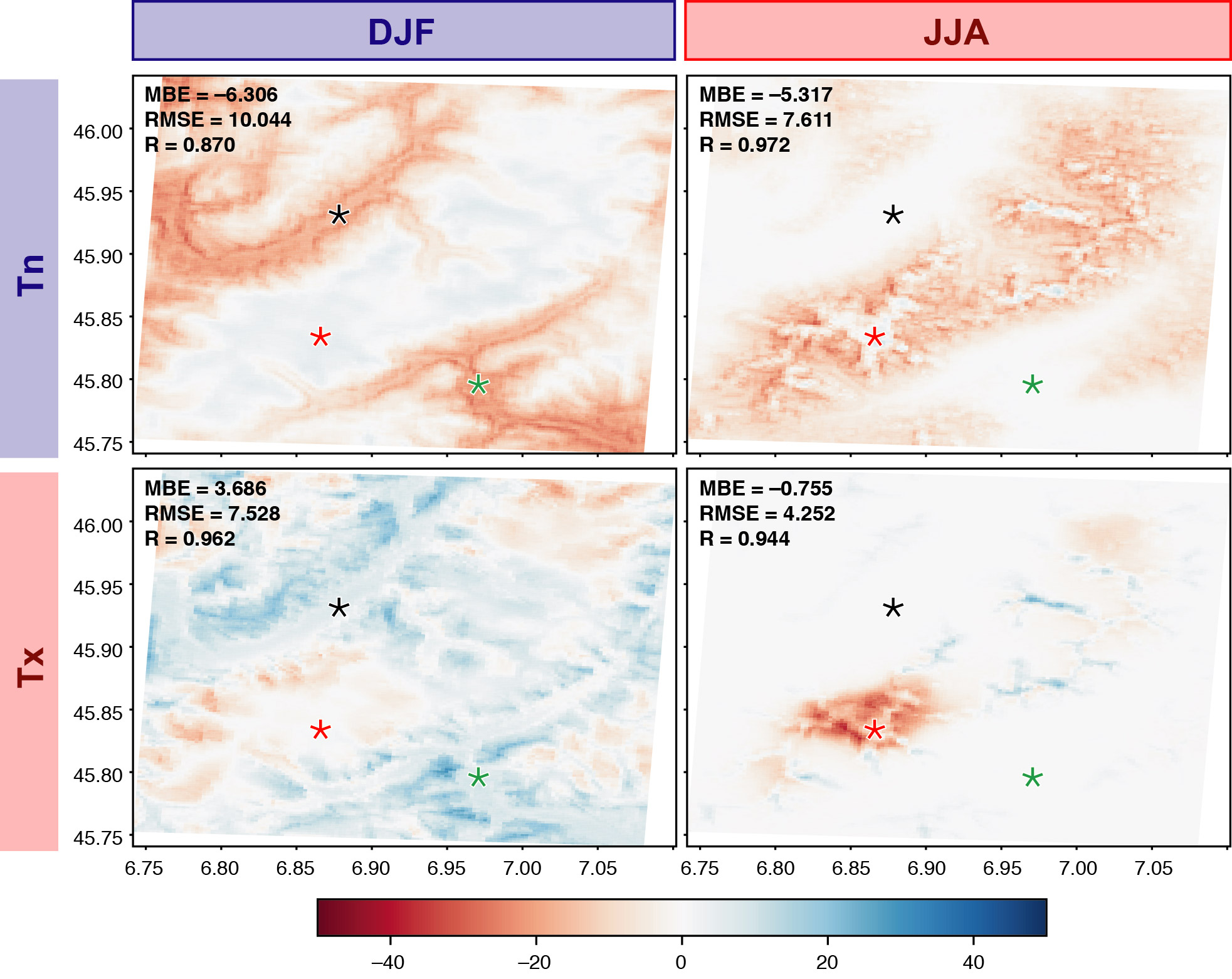


**Supplementary Figure 2 |** Biases (percentage points) of frost occurrence estimations of HIST simulations, obtained as the difference with spatially-interpolated observations, for both Tn and Tx and summer and winter seasons. Associated error estimates are labelled in the figure: spatially-mean bias error (MBE), root-mean-square error (RMSE) and spatial correlation between both fields (R).

**2. Winter frost frequency**

The main paper presents time evolutions in summer frost (**Figs. 4 and 5**). These analyses are here duplicated and shown for the winter season (**Supp. Figs. 4 and 5**). Once again the main changes occur close to the 0°C isotherm, which implies thus a general upward shift compared to the results obtained for the summer season.

In winter, the main changes in morning frost occurrence concern the bottom of the valleys (Chamonix and Courmayeur, **Supp. Fig. 4**), that is, elevations below 2000m asl (**Supp. Fig. 5**). There, the magnitude of the changes reach –8-10pp under RCP2.6 for both the mid- and late-century, showing that this scenario simulates roughly stationary climate conditions during the latest decades of the century. Changes are much larger for RCP8.5 for the mid-century horizon (–15pp), and even more dramatically for the late century (–30pp). Considering current frost frequency of about 75-80% in DJF in the morning (**Fig. 2**), this indicates that mountain stations like Chamonix or Courmayeur should experience freezing temperatures about one day out of two (50%) by the end of the century, under the most pessimistic scenario.

During the warmer afternoon hours, the 0°C isotherm reaches elevations close to 2000-2500m asl. This altitudinal range also concentrates the largest drop in frost frequency (**Supp. Figs. 4 and 5**). While the magnitude of the changes remains rather weak according to RCP2.6 projections (–10-12pp for the mid- and late century periods), it is already of about –18-20pp in 2036-65 and even –30-35pp in 2070-2100 under RCP8.5. This leads to an average frost frequency of about 25-40% there in the late century (instead of 60-70% under current climate conditions, **Fig. 2**).

These evolutions imply huge changes in the snow cover, and winter sport potentialities. In the valleys, rain could be much more frequent, and snow cover much more intermittent. Ski facilities should include the risk for defrost and lack of continuous snow for elevations up to 2500-3000m, calling for a radical change in the repartition of winter sport facilities compared to existing ones. Of course, these changes would be much reduced under an optimistic emission scenario.


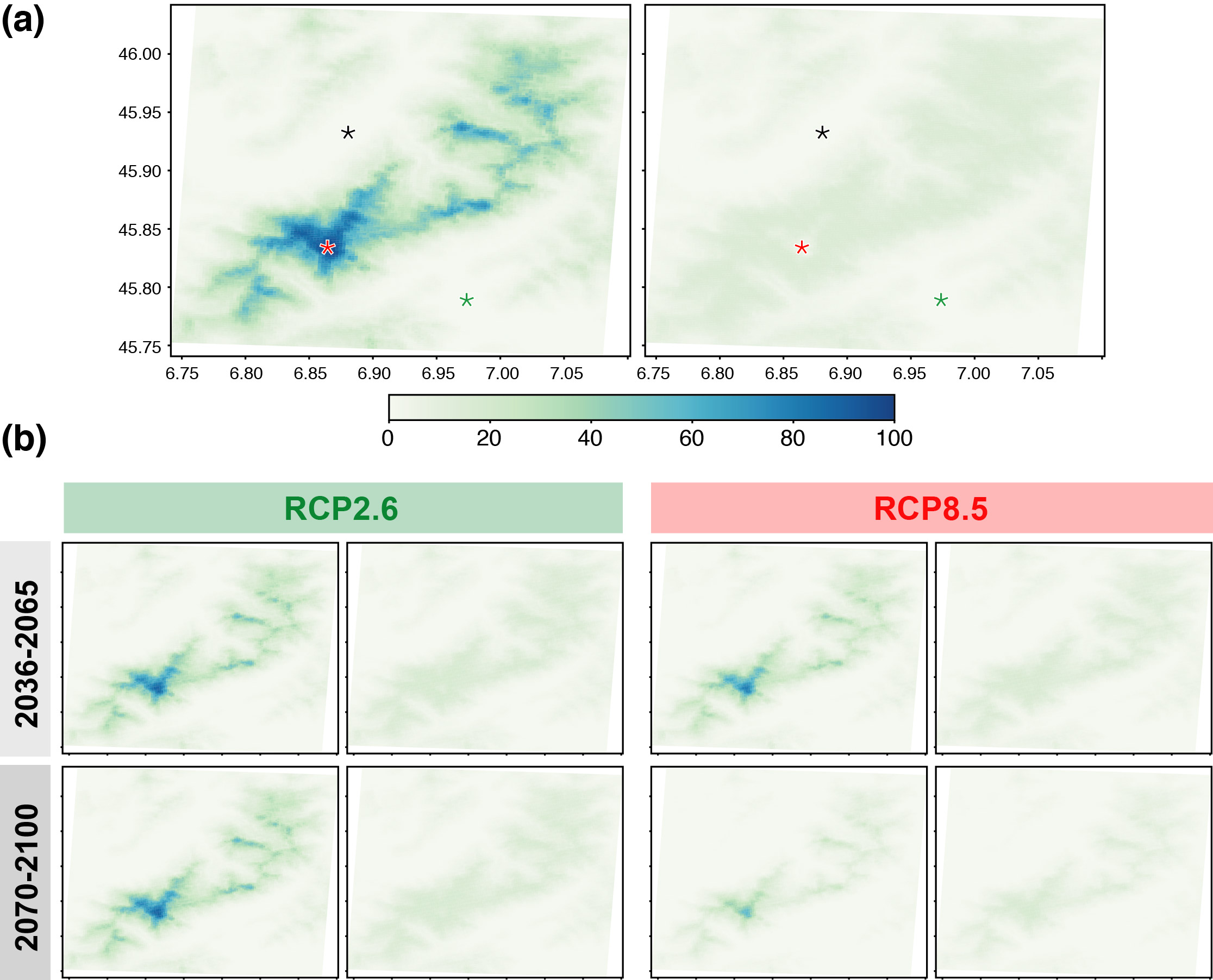


**Supplementary Figure 3 |** (a) Frost occurrence (%) for Tx and HIST simulations simulated by the CMIP5 multi-model ensemble mean (left-hand panel), and associated uncertainties computed as the inter-model standard deviation (right-hand panel), period JJA 1970-2000. (b) The same for RCP2.6 and RCP8.5 for mud-century and late century periods.


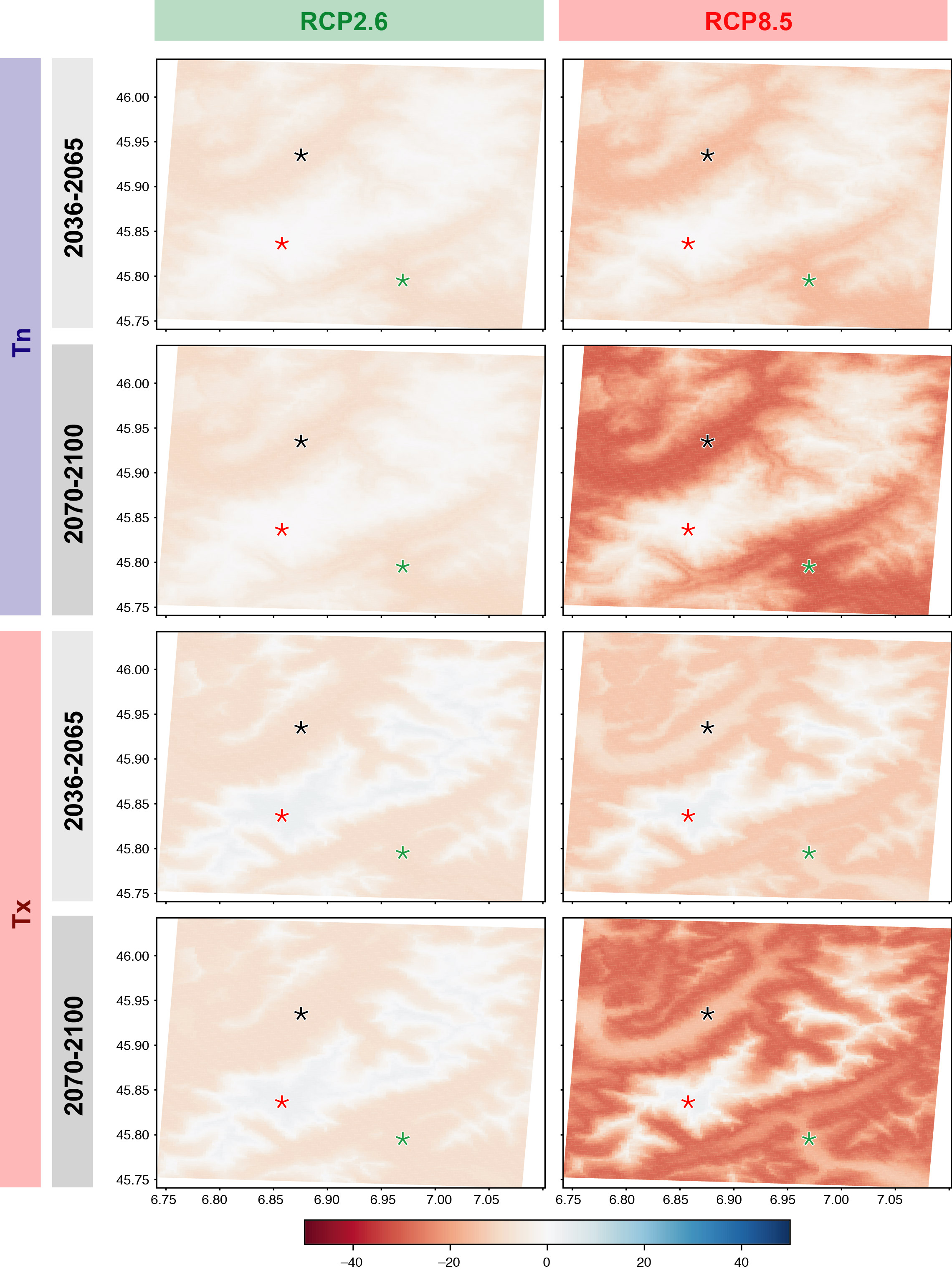


**Supplementary Figure 4 |** As Fig. 4 but for the winter season.


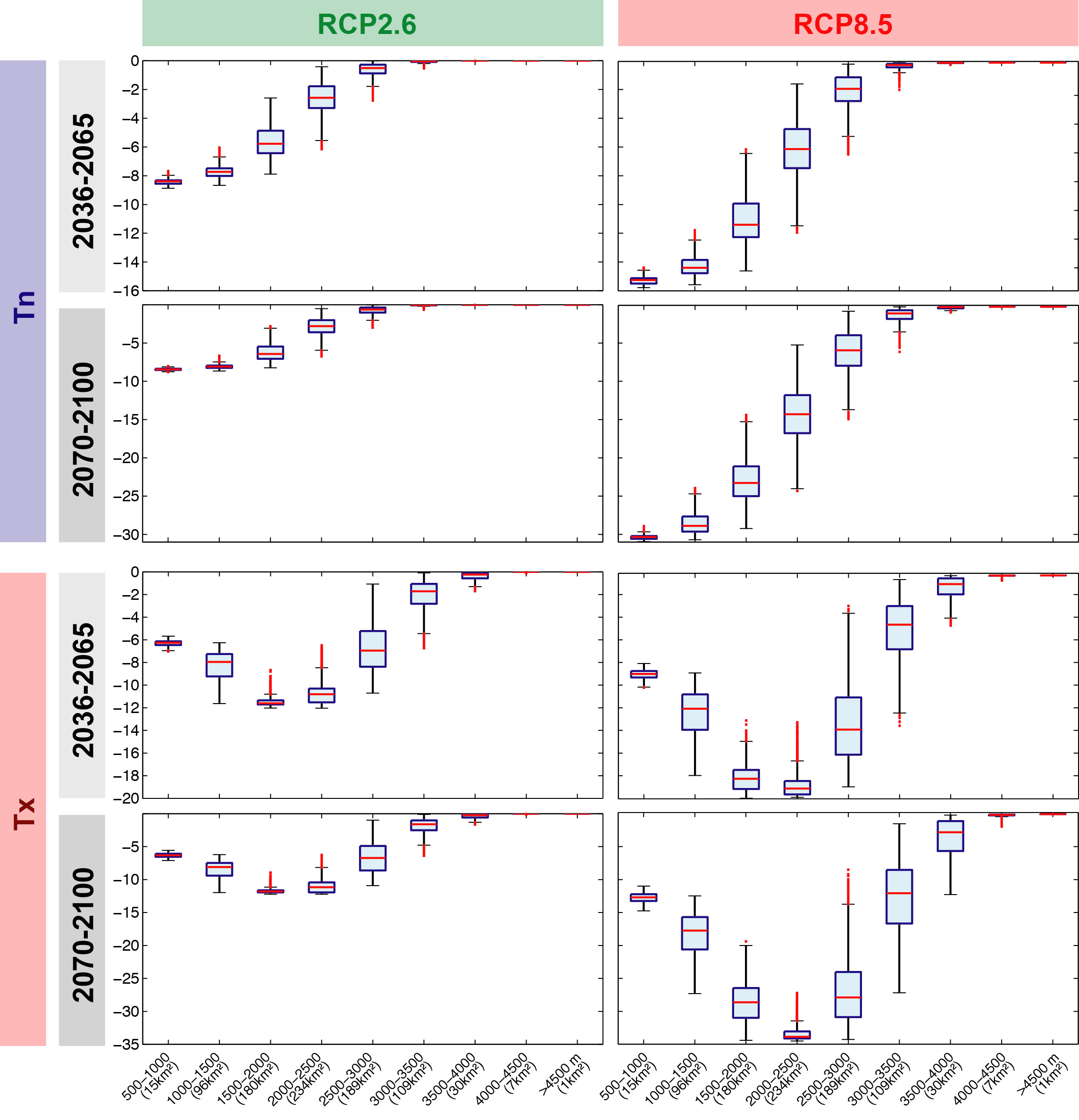


**Supplementary Figure 5 |** As Fig. 5 but for the winter season.

**Supplementary References**

1. Intergovernmental Panel on Climate Change. *Climate Change 2013: The Physical Science Basis. Contribution of Working Group I to the Fifth Assessment Report of the Intergovernmental Panel on Climate Change*. (2013).

2. Leduc, M., Laprise, R., de Elía, R. & Šeparović, L. Is institutional democracy a good proxy for model independence? *J. Clim.* **in press,** (2016).

3. Joly, D. *et al.* Geomatic downscaling of temperatures in the Mont Blanc massif. *Int. J. Climatol.* **38,** 1846–1863 (2018).

4. Nigrelli, G., Fratianni, S., Zampollo, A., Turconi, L. & Chiarle, M. The altitudinal temperature lapse rates applied to high elevation rockfalls studies in the Western European Alps. *Theor. Appl. Climatol.* **131,** 1479–1491 (2018).

5. Joly, D., Bois, B. & Zaksek, K. Rank-Ordering of Topographic Variables Correlated with Temperature. *Atmos. Clim. Sci.* **2,** 139–147 (2012).

6. von Storch, H., Zorita, E. & Cubasch, U. Downscaling of Global Climate Change Estimates to Regional Scales: An Application to Iberian Rainfall in Wintertime. *J. Clim.* **6,** 1161–1171 (1993).

7. Rogelj, J., Meinshausen, M. & Knutti, R. Global warming under old and new scenarios using IPCC climate sensitivity range estimates. *Nat. Clim. Chang.* **2,** 248 (2012).

8. Roe, G. H. & Baker, M. B. Why Is Climate Sensitivity So Unpredictable? *Science (80-. ).* **318,** 629 LP-632 (2007).

9. Hansen, J. *et al.* Climate Sensitivity: Analysis of Feedback Mechanisms. *Climate Processes and Climate Sensitivity* (17307BC). doi:doi:10.1029/GM029p0130

10. Jacob, D. *et al.* EURO-CORDEX: new high-resolution climate change projections for European impact research. *Reg. Environ. Chang.* **14,** 563–578 (2014).
